# Effects of Anesthetic Management on Early Postoperative Recovery, Hemodynamics and Pain After Supratentorial Craniotomy

**DOI:** 10.14740/jocmr2256w

**Published:** 2015-08-23

**Authors:** Eugenia Ayrian, Alan David Kaye, Chelsia L. Varner, Carolina Guerra, Nalini Vadivelu, Richard D. Urman, Vladimir Zelman, Philip D. Lumb, Giovanni Rosa, Federico Bilotta

**Affiliations:** a Department of Anesthesiology, Keck School of Medicine of the University of Southern California, Los Angeles, CA, USA; b Department of Anesthesiology, Critical Care and Pain Medicine, “Sapienza” University of Rome, Rome, Italy; c Department of Anesthesiology, Louisiana State University Health Sciences Center, New Orleans, LA 70112, USA; d Department of Anesthesiology, Yale University School of Medicine, New Haven, CT, USA; e Department of Anesthesiology, Perioperative and Pain Medicine, Brigham and Women’s Hospital/Harvard Medical School, Boston, MA 02115, USA; f Department of Anaesthesiology, Critical Care and Pain Medicine, “Sapienza” University of Rome, Policlinico Umberto I, Rome, Italy

**Keywords:** Neuroanesthesia, Postoperative recovery, Supratentorial, Craniotomy, Anesthesia, Cognitive recovery

## Abstract

Various clinical trials have assessed how intraoperative anesthetics can affect early recovery, hemodynamics and nociception after supratentorial craniotomy. Whether or not the difference in recovery pattern differs in a meaningful way with anesthetic choice is controversial. This review examines and compares different anesthetics with respect to wake-up time, hemodynamics, respiration, cognitive recovery, pain, nausea and vomiting, and shivering. When comparing inhalational anesthetics to intravenous anesthetics, either regimen produces similar recovery results. Newer shorter acting agents accelerate the process of emergence and extubation. A balanced inhalational/intravenous anesthetic could be desirable for patients with normal intracranial pressure, while total intravenous anesthesia could be beneficial for patients with elevated intracranial pressure. Comparison of inhalational anesthetics shows all appropriate for rapid emergence, decreasing time to extubation, and cognitive recovery. Comparison of opioids demonstrates similar awakening and extubation time if the infusion of longer acting opioids was ended at the appropriate time. Administration of local anesthetics into the skin, and addition of corticosteroids, NSAIDs, COX-2 inhibitors, and PCA therapy postoperatively provided superior analgesia. It is also important to emphasize the possibility of long-term effects of anesthetics on cognitive function. More research is warranted to develop best practices strategies for the future that are evidence-based.

## Introduction

Early post-operative recovery after supratentorial craniotomy is critically important for patients and highly desired by surgeons and anesthesiologists [1-3]. Low return to consciousness can make a neurological examination more difficult, add to diagnostic and/or therapeutic interventions, and predispose to respiratory complications. Various clinical trials have assessed how anesthetics administered intraoperatively can affect early postoperative recovery, hemodynamics and nociception.

Whether or not changes in recovery pattern differ significantly with anesthetic agent or technique remains controversial. Though choice of anesthetic agent largely reflects the way each clinician is taught how to deliver a neuroanesthetic, there are varying affects of each anesthetic with regard to intracranial elastance (compliance), control of cerebral blood flow and metabolism, brain protection, early neurological assessment, and hemodynamically stable emergence. Paramount to understanding the role of any anesthetic on each of these parameters in a patient with a supratentorial tumor, the clinician needs to appreciate that the cranial compartment is incompressible and the volume inside the cranium is a fixed volume.

At some point in tumor growth, this can significantly affect recovery and patient outcome. This concept, termed the Munro-Kellie Doctrine, dictates that the cranium and its constituents, e.g. blood, cerebral spinal fluid, and brain tissue, create a state of volume equilibrium, such that any increase in volume of one of the cranial constituents must be compensated by a decrease in volume of another. Adverse consequences of increasing intracranial volume can include brain herniation, cerebral ischemia, poor surgical exposure, and/or retractor-induced ischemia.

Basic science investigation has previously resulted in findings suggestive of a beneficial role of inhalational agents when compared to intravenous anesthetics in different animal models [4], demonstrating improved outcome in rats with inhalational agent versus intravenous agents, such as ketamine or fentanyl with near-complete but not complete cerebral ischemia as measured by percent of dead neurons. Hoffman and colleagues demonstrated enhancement of brain tissue oxygen levels in dogs with inhalational agents [5]. How translatable these results are for humans remains unclear at present.

The postoperative quality recovery scale (PQRS) has been based on domains such as physiology (hemodynamics and respiration), nociception (pain, nausea and vomiting), and cognitive recovery [2]. Therefore, in the present investigation to best ascertain if one technique or anesthetic agent possesses an advantage for patients with supratentorial tumor, we chose to complete a detailed literature review and to examine and to compare anesthetics with respect to wake-up time, hemodynamics, respiration, cognitive recovery, pain, nausea and vomiting, and shivering.

## Anesthetics and Early Post-Anesthesia Recovery

Early post-anesthesia recovery is a critical phase after surgery for supratentorial brain lesions and is considered to be a measure for anesthesia quality [1, 2, 6-9]. During early post-anesthesia recovery, patients should be in a stable physiologic state with regard to arterial blood pressure, heart rate, and respiratory rate, to minimize the risk for early postoperative complications. Recovery of cognitive abilities to allow a prompt clinical diagnosis of any potential postsurgical complications, such as an intracranial bleed, is equally important [3, 8, 10].

The definitive criterion for quality of recovery has not been fully established [1]. Rapid return to a level of consciousness that allows immediate neurologic assessment has previously been used [1, 2]. A review of the literature shows several quality of recovery parameters used in clinical trials comparing different anesthetic techniques. Examples include time to emergence (completion of dressings to extubation) [10-21], early versus late emergence [13, 16] (less versus greater than 15 min), time to eyes opening [11, 17], time to respond to commands [7, 19], achievement of Aldrete score of 9 or higher [7, 11-13, 22], and scoring on the Glasgow coma scale (GCS) [22, 23] and visual analogue scale (VAS) [13]. Short orientation memory concentration test (SOMCT) was the only test used in the studies to evaluate patients’ level of alertness [15, 21]. Other complex emergency assessment tests which are used for evaluation of patients undergoing outpatient surgeries are irrelevant in a neurosurgical population, where only simple neurologic evaluation is needed immediately after surgery [12].

In this section, published studies, which evaluate the effects of different strategies on early postoperative recovery after craniotomy are presented and categorized into three groups: 1) balanced inhalational versus total intravenous anesthesia; 2) different inhalational types of anesthesia; 3) different types of intravenous opioids.

### Balanced inhalational versus total intravenous anesthesia

In only one previous study, when propofol was compared to thiopental/isoflurane, during the first 20 - 30 min of recovery, the GCS score was significantly higher and postoperative recovery was faster in patients which received propofol, suggesting improved recovery profile. Eye opening, response to commands, extubation, speech, and time/space orientation were the factors which defined recovery [24]. This was the only study that included thiopental in its regimen. All subsequent recovery studies have been performed with propofol as an intravenous anesthetic. These results were not confirmed with subsequent studies that compared several anesthetics. Despite the fact that some studies showed slightly earlier extubation time [12, 24], quicker return to the level of consciousness score of ≤ 2 [11-12], and faster achievement of the Aldrete score ≥ 9 (Table 1) [12], no statistical or clinical significance was found in spite of treatment assignment (Table 2) [11-14, 16, 17, 23, 24]. The early extubation time (< 15 min) was similar in all groups [13, 16], which was clinically more important. Shorter acting anesthetics have helped to speed up the process of recovery [25], but thus far, no study demonstrated major outcome differences when total intravenous or volatile-based anesthetics for supratentorial craniotomies were compared [10, 11, 17, 18, 25-28].

**Table 1:** The Aldrete Score [12]

Activity	
2	Able to move spontaneously or on command four extremities
1	Able to move voluntarily or on command two extremities
0	Unable to move any extremities
Respiration	
2	Able to deep breath and cough freely
1	Dyspnea, shallow or limited breathing
0	Apneic
Circulation	
2	BP + 20 mm Hg of pre-sedation level
1	BP + 20 - 50 mm Hg of pre-sedation level
0	BP + 50 mm Hg of pre-sedation level
Consciousness	
2	Fully awake
1	Arousable on calling
0	Not responding
Skin color	
2	Normal
1	Pale, dusky, blotchy, jaundiced, other
0	Cyanotic

**Table 2:** Early Postoperative Recovery and Effects of Balanced Inhalational Versus Total Intravenous Anesthesia [11-14, 16, 17, 23, 24]

Studies	Drugs	Clinical variables	Results
Lauta et al, 2010 [11]	Sevoflurne/remifentanil vs. propofol/remifentanil	Aldrete score TEO ET	No clinical differences
Ravussin et al, 1991 [24]	Thiopental/isoflurane vs. propofol	GSC	Propofol group had higher GSC score in early recovery
Todd et al, 1993 [12]	Propofol/fentanyl vs. isoflurane/nitrous oxide vs. fentanyl/nitrous oxide	ET Time to commands Aldrete score	No statistical difference
Talke et al, 2002 [13]	Propofol vs. isoflurane vs. propofol/isoflurane	ET TEO Aldrete score VAS	No statistical differences
Magni et al, 2005 [14]	Sevoflurane/fentanyl vs. propofol/remifentanil	ET SOMCT	No statistical differences
Citerio et al, 2012 [23]	Sevoflurane/fentanyl vs. sevoflurane/remifentanil vs. propofol/remifentanil	Aldrete score	No statistical difference
Bhagat et al, 2008 [16]	At time of dural closure bolus of propofol vs. fentanyl vs. isoflurane	Time to emergence (dressing completion to extubation) Early emergence (< 15 min) GCS	Propofol > isoflurane > fentanyl 6 min > 5 min > 4 min (P = 0.0008) - not clinically significant No statistical difference
Sneyd et al, 2005 [17]	Propofol/remifentanil vs. sevoflurane/remifentanil	ET TEO Obey commands	No clinical differences

ET: extubation time; TEO: time to eyes opening; GCS: Glasgow coma scale; VAS: visual analogue scale; SOMCT: short orientation memory concentration test.

Therefore, according to the available clinical evidence, the use of balanced inhalational or total intravenous anesthesia is equally effective with regard to the timing of early post-anesthesia recovery after supratentorial craniotomies.

### Effects of different types of inhalational anesthesia

In patients undergoing supratentorial craniotomy, desflurane provides shorter time to extubation (2 min versus 6 min) [14] and recovery time, and earlier post-anesthesia cognitive recovery than sevoflurane (P < 0.005) [14, 29]. This should not be surprising, given that desflurane has lower solubility, which provides for more rapid emergence. Nevertheless, in both studies, cognitive impairment was limited to the first 15 min after extubation [14] or achieving Aldrete score equal or more than 9 [29], and within 30 min, it returned to normal. Despite the fact that rapid recovery of patient’s cognitive function is desirable, it is apparent that the impact of these differences on clinical practice is trivial [14].

### Effects of different types of intravenous opioids

The most commonly used opioids for supratentorial craniotomies are remifentanil, fentanyl, and sufentanil (Table 3) [7, 10, 18-23, 30]. Some of the studies comparing remifentanil to fentanyl have shown that the immediate recovery was faster with remifentanil. For example, in the remifentanil group, a higher percentage of patients showed normal recovery score (alert, oriented, able to follow commands, modified Aldrete score of 9 - 10) 10 min after surgery (P = 0.005) [23], an Aldrete score ≥ 9 was achieved faster (8.6 min versus 14.6 min, P < 0.001) [7], and GCS scores were highest immediately after extubation [22]. Remifentanil also required lower MAC of isoflurane throughout the procedure, less propofol was used [12], and naloxone was not required after surgery [24]. Neurosurgeons, who were blinded to the treatment group, favored the use of remifentanil for the patient comfort (P = 0.04), the level of consciousness (P = 0.02) and overall quality of emergence (P < 0.001) [10, 22]. Nevertheless, the difference in recovery score was eliminated between the groups (P = 0.27) 20 min after surgery [19].

**Table 3:** Early Postoperative Recovery and Effects of Different Types of Intravenous Opioids [7, 10, 18-23, 30]

Studies	Drugs	Clinical variables	Results
Guy et al, 1997 [10]	Remifentanil vs. fentanyl	ET Quality of recovery	No statistical difference Low recovery score within 60 min more in fentanyl group Naloxone use more in fentanyl group
Citerio et al, 2012 [23]	Sevoflurane/remifentanil vs. sevoflurane/fentanyl vs. propofol/remifentanil	Aldrete score	No statistical difference
Gerlach et al, 2003 [18]	Remifentanil/propofol vs. sufentanil IV pushes/propofol	ET	Remifentanil group had earlier ET 6.4 min vs. 14.3 min (P = 0.003)
Balakrishnan et al, 2000 [19]	Remifentanil vs. fentanyl	ET (P = 0.04) Obay commands (P < 0.001) ET (P = 0.04) Recovery in 10 min (P = 0.005) Recovery in 20 min	Better in remifentanil group No statistical difference
Van der Zwan et al, 2005 [22]	Remifentanil/piritramide vs. fentanyl	GCS Aldrete score	No statistical difference
Del Gaudio et al, 2006 [7]	Remifentanil vs. fentanyl	ET Obey commands Orientationx3 Aldrete score	Remifentanil group faster (P < 0.001)
Djian et al, 2006 [20]	Remifentanil vs. sufentanil	ET	No statistical difference
Bilotta et al, 2007 [21]	Remifentanil vs. sufentanil	ET SOMCT	No statistical difference Higher in remifentanil group (P < 0.0001)
From et al, 1990 [30]	Alfentanil vs. fentanyl vs. sufentanil	Alertness	More in alfentanil group

ET: extubation time; GCS: Glasgow coma scale; SOMCT: short orientation memory concentration test.

The prospective NeuroMorfeo study on 380 patients concluded that there was no difference in time to reach an Aldrete score ≥ 9 between remifentanil and fentanyl groups [23]. Another study comparing early postoperative recovery after use of fentanyl versus remifentanil in supratentorial craniotomies showed no differences in emergence time and in the timing to achieve an Aldrete score ≥ 9 [22]. When fentanyl was used as a continuous infusion, the extubation time was identical to remifentanil, which could be explained by an earlier interruption of the fentanyl infusion [22]. This supports the conclusion that opioid infusions can be predictably used if the infusions are ended at the appropriate time.

When comparing remifentanil to sufentanil, only one of the experiments showed that patients in the remifentanil group were extubated earlier (6.4 min versus 14.3 min; P = 0.003) [18] and their SOMCT scores were significantly higher in the remifentanil group [21].

More recent studies have shown that extubation time and the time to achieve an Aldrete score ≥ 9 were similar in both groups [20, 21]. In one of these studies, the sufentanil drip was stopped earlier in the course of the procedure than the remifentanil drip [21]. Therefore, with proper timing for discontinuing the infusion, one can achieve the same extubation and recovery time.

An earlier study comparing alfentanil to fentanyl and sufentanil did not show any significant difference in the level of conciseness after anesthesia, although alfentanil-treated patients appeared more alert 30 min after arrival to the recovery area in comparison to the other two groups [30].

According to available evidence, the selection of balanced inhalational anesthesia, total intravenous anesthesia, or the use of short-acting anesthetics when appropriately discontinued, does not appear to affect the extubation time and the time necessary to reach an Aldrete score ≥ 9 at the level [31] that would be considered clinically significant.

### Anesthetics and cognitive recovery

There has been a growing interest in determining whether there is a relationship between anesthesia and the onset and progression of chronic neurodegenerative disorders, including Alzheimer’s disease. Theoretically, the factors responsible for the development of chronic neurodegenerative disorders and persistent post-anesthetic cognitive dysfunction may overlap [32]. Rapid recovery is warranted and offers numerous advantages in these patients, such as evaluation of the neurological status and detection of early surgical complications [30]. Cognitive recovery has become one of the most important variables investigated in clinical trials for comparing different anesthetic strategies [1, 30]. Over two decades ago, researchers were eager to compare cognitive recovery from different anesthetics. They were able to demonstrate that propofol infusion was followed by a faster recovery of cerebral functions (e.g. faster eye opening, response to commands, extubation, speech, and time/space orientation) when compared to the longer acting thiopental-isoflurane anesthesia [24]. In recent years, studies for postoperative cognitive dysfunction after craniotomy have concentrated on comparison of different types of anesthetics such as inhalational anesthetics and opioids, in addition to other agents such as dexmedetomidine [21, 32]. In a study that compared remifentanil-propofol and sufentanil-propofol anesthetic, patients in the remifentanil group experienced significantly faster cognitive recovery and better results on the SOMCT [21]. Comparison of remifentanil to dexmedetomidine revealed that patients receiving dexmedetomidine demonstrate a slower recovery of cerebral function (extubation time, response to verbal commands, and time for orientation) (P < 0.05). Those receiving remifentanil, on the other hand, require earlier demand for postoperative analgesics [33].

In summary, the use of shorter acting medications, such as propofol and remifentanil, can lead to the faster recovery of cerebral functions, when compared to the longer acting anesthetics. Remifentanil also allows the earliest cognitive recovery when compared to other intravenous agents. Nevertheless, fentanyl, sufentanil, and dexmedetomidine are equivalently appropriate for supratentorial craniotomies [19, 21, 33].

### Anesthetics and hemodynamic recovery

Arterial hypertension is one of the most common complications after craniotomy for supratentorial lesions [31]. The blood flow velocity may increase 60% above preoperative level during extubation and may remain elevated for 30 - 60 min after extubation [34]. Acute postoperative arterial hypertension may lead to an increase in intracranial pressure, development of cerebral hyperemia, vasogenic edema, and can potentially advance into intracranial hemorrhage. Intracranial hemorrhage independently can lead to prolonged hospital stay and can possibly become a fatal complication [35]. The etiology of arterial hypertension is multifactorial [36]. It can be induced by pain and arousal [36]. It can also be referred to physiological changes and to effects of medications used throughout the procedure [21, 34, 36]. The arterial hypertension can be explained by surgical stress itself and, therefore, catecholamine release and activation of the renin-angiotensin-aldosterone system, which causes stimulation of the sympathetic system and amplification of the vasoconstrictive effect of the catecholamines [34, 36].

The definition of hypertension after supratentorial craniotomies varies in different studies. Most commonly it has been defined as mean arterial pressure (MAP) exceeding the baseline by 20% [21, 29, 36], 25% [30], or rising above 100 mm Hg for more than 1 min [17]. One study described hypertension in terms of systolic blood pressure (SBP) exceeding the baseline by 20% [37]. The overall incidence of postoperative hypertension after supratentorial craniotomies runs in 30-35% range [21, 31]. One of the studies, which reviewed 12 patients after supratentorial craniotomy, defined that 50% of patients experienced 20% increase of MAP over the baseline postoperatively [36]. Another study of 60 patients demonstrated as much as 82% of them experience the 20% increase in SBP immediately after extubation [37]. Treatment with esmolol [37], labetalol, or hydralazine [17] was implemented when appropriate.

There are many reasons contributing to postoperative hypertension after supratentorial craniotomies. In addition, drug-induced arterial hypertension can also arise after the use of short acting opioids (i.e. remifentanil) and some inhalational anesthetics (i.e. desflurane) [21, 29, 37]. Multiple clinical studies have focused on reduction of postoperative hemodynamic complications (Table 4) [7, 10-14, 16, 17, 19, 20, 24, 30, 38]. We have categorized them into three groups: 1) use of intravenous versus inhalational anesthetics, 2) effects of opioids, 3) antihypertensive medications.

**Table 4:** Studies Addressing Postoperative Hemodynamic Recovery [7, 10-14, 16, 17, 19, 20, 24, 30, 38]

Studies	Drugs	Clinical variable	Results
Lauta et al, 2010 [11]	Sevoflurane/remifentanil vs. propofol/remifentanil	HR MAP	No statistical difference Sevo/Remi - more hypotension episodes (P = 0.0167)
Ravussin et al, 1991 [24]	Thiopental/isoflurane vs. propofol	HR, MAP CPP	No statistical difference Lower in propofol group
Todd et al, 1993 [12]	Propofol/fentanyl vs. isoflurane/nitrous oxide vs. fentanyl/nitrous oxide	ICP MAP HR	Iso/N2O - higher ICP, HR, lower MAP The difference is not statistically significant
Guy et al, 1997 [10]	Remifentanil vs. fentanyl	SBP ICP, CPP	Higher in fentanyl group P = 0.004 No statistical difference
Talke et al, 2002 [13]	Propofol vs. isoflurane vs. propofol/isoflurane	BP, HR	No statistical difference
Magni et al, 2005 [14]	Sevoflurane/fentanyl vs. propofol/remifentanil	MAP: intraoperative hypotension; postoperative HTN HR	Remifentanil group had more hypotension intraoperative (P = 0.002), hypertension postoperative (P = 0.0046) No statistical difference
Bhagat et al, 2008 [16]	Propofol vs. fentanyl vs. isoflurane		Less esmolol use with fentanyl P = 0.001
Sneyd et al, 2005 [17]	Remifentanil/propofol vs. remifentanil/sevoflurane	MAP	Remi/Sevo - more hypotension episodes (P = 0.053)
Balakrishnan et al, 2000 [19]	Remifentanil vs. fentanyl	HR, MAP	No statistical difference
Del Gaudio et al, 2006 [7]	Remifentanil vs. fentanyl	HR, MAP	No statistical difference
Djian et al, 2006 [20]	Remifentanil vs. sufentanil	MAP HR	Remifentanil P = 0.037 better MAP, HR stability
From et al, 1990 [30]	Alfentanil vs. fentanyl vs. sufentanil	RR PH	Lower after extubation in Sufentanil group
Warner, 1996 [38]	Alfentanil vs. remifentanil	ICP MAP decrease	No statistical difference

### Use of intravenous versus inhalational anesthetics

Total intravenous anesthesia induces lower subdural intracranial pressure, higher cerebral perfusion pressure, and less pronounced cerebral swelling in comparison to inhalational anesthesia [39]. In terms of postoperative hemodynamic changes, the differences in blood pressure and heart rate are not statistically significant [11-13, 17, 24] with the exception of one study which concluded that remifentanil is associated with more frequent postoperative hypertension than sevoflurane/fentanyl (17% versus 40%, P = 0.0046) [15]. Most patients receive antihypertensive therapy postoperatively after supratentorial craniotomy [12]. One study concluded that labetalol was more frequently used in patients receiving propofol than isoflurane (P = 0.015) [13]. Nevertheless, in patients with normal intracranial pressure, inhalational anesthesia with sevoflurane appears to be as safe and appropriate as intravenous anesthesia during elective craniotomy for the removal of a supratentorial neoplasm [11, 25, 40].

It has been proposed that by delaying the process of recovery and extubation, the metabolic changes and therefore, postoperative hemodynamic changes, would be attenuated. Hemodynamic changes have been shown to be statistically insignificant after delayed extubation. Stress response was not eliminated, but prolonged with delaying extubation for 2 h [6]. Therefore, delayed recovery would not offset hemodynamic changes after supratentorial craniotomy.

Comparison between intravenous and inhalational anesthesia for supratentorial craniotomy reveals that despite minimal differences, short-term outcome in terms of hemodynamic stability is not affected in patients with normal intracranial pressure [11, 13, 15]. Therefore, it is possible that a combined inhalational/intravenous anesthetic could be a better option for patients with normal intracranial pressure during supratentorial craniotomies than pure intravenous anesthesia or inhalational anesthesia alone [40].

## Effects of Opioids

The effects of opioids with different half-life and potency (alfentanil, sufentanil, fentanyl, and remifentanil) on postoperative recovery after supratentorial craniotomy have been extensively studied without reporting major clinical differences [25, 30]. Most of the studies have not demonstrated a clear benefit, but have shown that the use of some of these drugs induce specific side effects. An intraoperative infusion of remifentanil demonstrated increased sympathetic tone, postoperative hyperalgesia, arterial hypertension, and tachycardia in the early postoperative period [7, 10, 21, 37]. SBP postoperatively was significantly higher in patients after remifentanil use in comparison to fentanyl (P = 0.001) [7, 10] or sufentanil [20]. Labetalol, hydralazine, and esmolol drip were used more commonly with remifentanil than with fentanyl [10, 19]. It has been shown that despite injection of local anesthetics into the wound, and postoperative treatment with morphine, antihypertensive medications were used more frequently after remifentanil use [10, 20]. It remains unclear whether acute tolerance to remifentanil, rapid elimination, or withdrawal syndrome after prolonged infusion results in increases in sympathetic tone after supratentorial craniotomy [29].

In contrary, there has been a study on 105 patients that failed to prove remifentanil caused postoperative hypertension more often than fentanyl. This finding could have been explained by the use of “transitional” analgesia with fentanyl, ketorolac, or morphine, which was allowed for the patients in remifentanil group before emergence from anesthesia [19]. Further, the main goal of the study was to evaluate the time for awakening, but not hemodynamics after remifentanil versus fentanyl use. The evaluation of previously recorded blood pressure pattern did not show any statistical differences between two groups, which could have resulted from an inadequate sample size or other bias [19].

Systemic hypertension during emergence on its own may contribute to the development of postoperative hematomas [19, 36]. Fentanyl or sufentanil infusions lead to lower average MAP postoperatively in comparison to remifentanil infusion [26, 28]. It has also been shown that administration of low-dose fentanyl at the time of dural closure leads to fewer occurrences of postoperative hypertension than with the use of propofol and isoflurane alone [17].

Therefore, alfentanil, sufentanil, fentanyl and remifentanil provide similar postoperative hemodynamics and are all appropriate for a hemodynamically stable awakening. The hypertension associated with discontinuation of a remifentanil infusion can be managed with administration of numerous antihypertensive medications [37]. For example, recent study found that a continuous infusion of landiolol suppressed hyperdynamic responses to stimuli during anesthesia while maintaining arterial blood pressure and cerebral oxygen balance. The landiolol infusion did not affect recovery from anesthesia and incidence of PONV, but it reduced intraoperative requirement of fentanyl [41].

## Antihypertensive Medications

The administration of antihypertensive medications plays a significant role in the management of patients after supratentorial craniotomies [37, 42-46]. It is generally recommended that postoperative exacerbation of hypertension be avoided by preemptive therapy. β-receptor antagonists and calcium-channel blockers are the most commonly utilized medications for reduction of postoperative hypertension after craniotomies [37, 45, 46].

Esmolol can effectively treat tachycardia and hypertension during extubation and in the immediate postoperative period [37, 44]. Esmolol blunts the increase in cerebral blood flow without significant effect on intracranial pressure, because of lack of cerebral vasodilatation properties [44]. Labetalol can be safely used to treat arterial hypertension in patients undergoing supratentorial craniotomy. It preserves cerebral blood flow, autoregulation, and does not affect global or regional cerebral blood flow or cerebral metabolic oxygen consumption [47, 38]. Nicardipine is commonly used in these situations to treat acute arterial hypertension effectively. It decreases the deviation of regional cerebral blood flow from the mean cerebral blood flow and increases the total cerebral blood flow [46]. Blood pressure profile with nicardipine is similar to labetalol with marginally smaller incidence of failure [46].

Because arterial hypertension [48] is a potential complication after craniotomy for supratentorial lesion that can lead to an increased risk of early postoperative bleeding, its management is extremely important [35]. In total, balanced inhalational/intravenous anesthetic with opioid use (fentanyl, sufentanil or remifentanil) is appropriate for a hemodynamically stable awakening. Furthermore, the administration of antihypertensive medications plays a significant role in the management of these patients. It is desirable to avoid postoperative exacerbation of hypertension by preemptive therapy with β-receptor antagonists and calcium-channel blockers.

## Anesthetics and Postoperative Pain

Postoperative pain after craniotomy, compared to other surgical procedures such as lumbar laminectomy, is less intense and associated with lower opioids consumption [1, 49]. This is evident in the fact that patients use fewer opioids, and stay in the PACU for a shorter period of time after these procedures [49]. Sixty percent of patients undergoing neurosurgical procedures complain of pain after surgery, and two-thirds of them experience moderate to severe pain [50, 51]. It has been shown that postoperative pain after craniotomy is often poorly controlled, undertreated, and requires improved and individualized pain management [50, 52, 53]. Insufficient treatment of postoperative pain after supratentorial craniotomies can lead to decreased patient satisfaction and adverse physical and physiological outcomes [1]. There are two clinical challenges that need to be balanced when taking care of patients after craniotomy. Controlling hypertension and minimizing the occurrence of postoperative complications is vital; however, on the other hand, a speedy recovery from anesthesia enables the neurosurgeon to complete early neurologic examination and quickly detect possible complications [49, 50].

The intensity of pain immediately after craniotomy is multifactorial. Acute pain after craniotomy is mostly superficial, has somatic origin, and involves the scalp, pericranial muscles, and soft tissue [54]. Therefore, the surgical approach influences the severity of pain. Supratentorial craniotomies are associated with less pain than infratentorial procedures, which can be explained by the amount of muscle damage from the resection of the temporal and posterior cervical muscles [52, 54, 55]. Patients’ positioning, head fixation in the Mayfield clamp, and extreme head rotation with tension on neck muscles, nerves, and spinal cord does not appear to influence the incidence of postoperative pain [52]. Acute pain can also originate from manipulation of the dura mater, but the dura is not richly innervated with pain receptors [56]. Post-craniotomy pain inversely correlates to patients’ age [50, 52]. The probability of experiencing postoperative pain is reduced by 3% for each additional year of life; 63% of patients with no pain are older than 49 years [52]. It can be explained by higher pain tolerance in elderly patients secondary to a loss of pain perception and longer effect of analgesic agents secondary to altered pharmacokinetics and the age-related reduction of plasma proteins [52]. A significantly higher level of postoperative pain is reported in women and patients who are taking opioids preoperatively [50, 54, 55]. In 17.5-29.3% of cases, chronic headache persists for more than 2 - 3 months after supratentorial craniotomy [54, 56]. We intend to revise the role of different anesthetics in early postoperative pain management after supratentorial craniotomies (Table 5) [10, 11, 13, 18, 20, 22], which will be categorized into two groups: 1) role of perioperative opioid treatment in pain management after supratentorial craniotomy, 2) a multimodal approach to anesthesia with the use of local anesthetics and medications other than opioids.

**Table 5:** Summary of Studies Addressing Postoperative Pain [10, 11, 13, 18, 20, 22]

Studies	Drugs	Clinical variable - pain
Lauta et al, 2010 [11]	Sevoflurane/remifentanil vs. propofol/remifentanil	No statistical difference
Guy et al, 1997 [10]	Remifentanil vs. fentanyl	No statistical difference
Talke et al, 2002 [13]	Propofol vs. isoflurane vs. propofol/isoflurane	No statistical difference
Gerlach et al, 2003 [18]	Remifentanil/propofol vs. sufentanil/propofol	No statistical difference
Van der Zwan et al, 2005 [22]	Remifentanil/piritramide vs. fentanyl	Remifentanil - more pain postoperatively Not statistically significant
Djian et al, 2006 [20]	Remifentanil vs. sufentanil	Remifentanil - greater use of morphine postoperatively (P = 0.016)

### Opioid analgesia

The choice of opioids administered during supratentorial craniotomies is important with regard to the course and severity of postoperative pain [57]. Because remifentanil may cause hyperalgesia and increase analgesic requirements postoperatively [10, 52], patients who are managed by administration of remifentanil infusion may require preoperative administration of gabapentin, parecoxib, and lornoxicam [54, 58]. It has been shown that pretreatment with morphine or fentanyl [54] or a combination of remifentanil with a longer-acting opioid after dura closure has no advantage over the sole use of fentanyl [22]. Remifentanil has also been compared to longer acting opioids such as sufentanil, and they both have demonstrated adequate intraoperative analgesia for supratentorial craniotomies, but more frequently are associated with postoperative hypertension [21]. Other opioid use has been investigated as well. Morphine is a better alternative for postoperative analgesia than codeine; it is more effective than codeine beyond 60 min (P = 0.01) and requires fewer doses than codeine (P = 0.003) [53, 59]. Intraoperative morphine was found to reduce total postoperative analgesic rescue doses [51, 57]. Tramadol has the potential risk of seizures and nausea and vomiting and tends to be avoided for supratentorial craniotomies [54].

Therefore, we can conclude that remifentanil is a fast, predictable opioid, which can cause postoperative hyperalgesia and thus, should be supplemented by the use of other medications [10, 22, 52, 54]. Other opiates also provide adequate analgesia, and can be utilized for pain management in patients undergoing supratentorial craniotomy [21, 60]. The method of delivery of pain medications also has an impact on how well pain is managed after supratentorial craniotomies. Very often it is controlled by patient-controlled analgesia (PCA). Patients on PCA have a significantly lower pain score than patients on conventional as needed (“Pro re nata”, i.e. “upon request”: PRN) therapy; at the same time, patients on PCA receive a higher amount of opioids than the patients on PRN therapy. There is no difference in adverse events in both groups [61]. Therefore, patients on PCA are treated for pain more effectively than patients on PRN therapy [61].

### Non-opioid analgesia

A multimodal approach to analgesia can lead to more successful treatment of post-craniotomy pain. Injection of local anesthetic into the scalp can block the conduction of abundant sensory nerve impulses, prevent the response to head pin application and the incision [62], decrease the level of pain within the first hour after craniotomy, and provide more intra- and postoperative hemodynamic stability [51, 57]. The addition of cyclooxygenase-2 (COX-2) inhibitors to opioids provides better pain control postoperatively, reduces the amount of opioid intake and the occurrence of opioid-induced side effects, and encourages earlier patient mobilization and reduction of the total hospitalization cost [60]. However, these results were not supported in a recent study which concluded that parecoxib use is not clinically beneficial for postoperative pain management after supratentorial craniotomy [51, 59]. The addition of non-steroidal anti-inflammatory drugs (NSAIDs) to the opioid regimen leads to better pain control after surgery, but administration of NSAIDs alone is not adequate for treatment of postoperative pain after supratentorial craniotomy [53]. Nalbuphine, a synthetic opioid agonist-antagonist analgesic is chemically related to both the opioid analgesic, oxymorphone and the opioid antagonist, naloxone. The addition of nalbuphine to the opioid regimen gives better pain control after supratentorial craniotomy, especially within the first hour after surgery [54]. Gabapentin, a prophylactic anticonvulsant medication, significantly was demonstrated to reduce propofol, remifentanil, and morphine consumption. However, it tends to delay the extubation and increase the level of sedation postoperatively [58]. The risk for postoperative pain increases by 119% in the absence of corticosteroids use. It can be explained by inhibition of prostaglandin synthesis by corticosteroids, which leads to an anti-inflammatory effect and an increase in endorphine levels followed by mood alteration [52].

Thus, optimal pharmacological treatment should be based on multiple considerations. Pain relief should combine an administration of shorter and longer acting opioids, the injection of local anesthetics into the skin, and administration of corticosteroids during the procedure. In addition, the administration of other medications such as NSAIDs, COX-2 inhibitors, and placing patients on PCA therapy postoperatively may also provide superior analgesia.

## Conclusion

Anesthetics administered in the perioperative period can markedly affect the postoperative/post-anesthesia course and recovery. In this review article we have evaluated the relationship between anesthetics and postoperative/post-anesthesia recovery in patients who have undergone brain surgery for supratentorial lesions.

When comparing inhalational anesthetics to total intravenous anesthetics, either regimen produces similar results with regard to emergence and to extubation time, return of cognitive function immediately after anesthesia, hemodynamic stability, and PCO_2_ level. Newer shorter acting agents accelerate the process of emergence and extubation in these patients. A balanced inhalational/intravenous anesthetic could be desirable for patients with normal intracranial pressure, while total intravenous anesthetic could be beneficial for patients with elevated intracranial pressure. The comparison of different inhalational anesthetics showed that they were all appropriate for a rapid emergence, decreasing time to extubation, and cognitive recovery.

Additionally, the comparison of different opioids to each other demonstrated similar awakening and extubation time if the infusion of longer acting opioids was ended at the appropriate time. The use of remifentanil appeared to cause postoperative hypertension, which was safely managed with the administration of antihypertensive medications. All opioids provided adequate analgesia. Remifentanil, however, was shown to cause a hyperalgesic effect after its cessation and had to be supplemented by the use of longer acting opioids or other medications in postoperative period. Administration of local anesthetics into the skin, and addition of corticosteroids, NSAIDs, COX-2 inhibitors, and PCA therapy postoperatively provided superior analgesia.

Finally, it is important to emphasize the possibility of the long-term effects of anesthetics on cognitive function, as they can become a trigger for the occurrence of chronic neurodegenerative disorders, such as Alzheimer disease [53]. In patients undergoing craniotomy for supratentorial lesions, there are several options of anesthesia management which will provide better postoperative recovery, more stable hemodynamics, rapid emergence, and adequate pain relief. More research in these areas is warranted to develop best practices strategies for the future that are evidence-based.
